# Genetically unresolved case of Rauch-Steindl syndrome diagnosed by its wolf-hirschhorn associated DNA methylation episignature

**DOI:** 10.3389/fcell.2022.1022683

**Published:** 2022-12-15

**Authors:** Haley McConkey, Alexandre White-Brown, Jennifer Kerkhof, David Dyment, Bekim Sadikovic

**Affiliations:** ^1^ Verspeeten Clinical Genome Centre, London Health Sciences Centre, London, ON, Canada; ^2^ Department of Pathology and Laboratory Medicine, Western University, London, ON, Canada; ^3^ Children’s Hospital of Eastern Ontario Research Institute, University of Ottawa, Ottawa, ON, Canada; ^4^ Department of Genetics, Children’s Hospital of Eastern Ontario, Ottawa, ON, Canada

**Keywords:** epigenetics, DNA methylation, molecular diagnostics, neurodevelopmental disorders, episignature

## Abstract

Wolf-Hirschhorn syndrome (WHS) is caused by deletion of a critical region of the short arm of chromosome 4. Clinical features of WHS include distinct dysmorphic facial features, growth restriction, developmental delay, intellectual disability, epilepsy, and other malformations. The *NSD2* gene localizes within this critical region along with several other genes. Pathogenic variants in *NSD2* cause Rauch-Steindl (RAUST) syndrome. Clinical features of RAUST syndrome partially overlap with WHS, however epilepsy and the recognizable facial gestalt are not observed. Here, we report a case of a young boy who presented with developmental delay, dysmorphic features and short stature. After negative chromosomal microarray and whole exome sequencing, genomic DNA methylation episignature analysis was performed. Episignatures are sensitive and specific genome-wide DNA methylation patterns associated with a growing number of rare disorders. The patient was positive for the WHS episignature. Reanalysis of the patient’s exome data identified a previously undetected frameshift variant in *NSD2*, leading to a diagnosis of RAUST. This report demonstrates the clinical utility of DNA methylation episignature analysis for unresolved patients, and provides insight into the overlapping pathology between WHS and RAUST as demonstrated by the similarities in their genomic DNA methylation profiles.

## Introduction

Wolf-Hirschhorn syndrome (WHS; OMIM# 194790) is caused by partial deletion of the short arm of chromosome 4 (Hirschhorn et al., 1965; Wolf et al., 1965). Clinical features include a recognizable facial gestalt often described as resembling a “Greek warrior helmet”, in addition to global developmental delay, intellectual disability, epilepsy, growth restriction, hypotonia and congenital heart malformations (Battaglia et al., 2001; Zollino et al., 2008). In early patients with a severe phenotype, large 4p deletions were identified and associated with the syndrome, however, smaller deletions have since been identified in the 4p16.3 region and are associated with a mild or atypical WHS phenotype (Zollino et al., 2008). While the size of the 4p deletion varies between patients, it is established that the recognizable WHS phenotype is due to hemizygosity of the 4p16.3 region (Zollino et al., 2008), and not due to the action of a single gene.

WHS exhibits a unique genome-wide methylation pattern, also called an episignature, that can be assessed in a patient’s blood sample (Levy et al., 2022). Episignatures can be used as sensitive and specific biomarkers for patients with ambiguous clinical or genetic findings (Aref-Eshghi et al., 2017; Aref-Eshghi et al., 2018; Aref-Eshghi et al., 2019a; Aref-Eshghi et al., 2020; Sadikovic et al., 2021; Kerkhof et al., 2022). They have also been proven useful in screening genetics naïve patients, as well as in the reclassification of variants of unknown significance (Sadikovic et al., 2021), including in disorders where the causative variant interrupts the function of an epigenetic machinery protein (also known as chromatinopathies) (Kerkhof et al., 2022). Currently, there are 57 clinically validated episignatures associated with 65 genetic syndromes (Levy et al., 2022). These DNA methylation patterns can be protein complex, gene, sub-gene, protein domain, and even single nucleotide, specific.

*NSD2* is one of several genes located within the critical 4p16 region and when mutated results in an overlapping phenotype of intrauterine growth restriction, growth restriction, hypotonia, and intellectual disability known as Rauch-Steindl syndrome (Zanoni et al., 2021) (RAUST; OMIM# 619695). Congenital malformations are present in some patients with RAUST, but at a lesser frequency than observed in WHS. Intractable seizures and the “Greek helmet appearance” are not a feature for those with RAUST syndrome (Zanoni et al., 2021). *NSD2* (OMIM# 602952) is expressed early in development and encodes a SET domain-containing histone methyltransferase that demethylates histone 3 at lysine 36, a histone modification associated with transcriptional activation.

Here we describe the case of a young boy presenting with syndromic intellectual disability and developmental delay with short stature, as well as dysmorphic features. Chromosomal microarray for this patient was negative and no compelling candidate variants in neurodevelopmental syndrome genes were detected by singleton exome sequencing. Subsequent DNA methylation episignature analysis was positive for the WHS methylation profile, resulting in a re-evaluation of the exome sequencing data, on a research basis. The re-assessment resulted in the identification of a previously missed single nucleotide likely pathogenic deletion variant in *NSD2* resulting the diagnosis of RAUST.

## Materials and methods

Methylation analysis was performed with the clinically validated EpiSign^TM^ assay as previously described (Aref-Eshghi et al., 2019a; Aref-Eshghi et al., 2019b; Aref-Eshghi et al., 2020; Sadikovic et al., 2021; Kerkhof et al., 2022). Briefly, methylated and unmethylated signal intensity generated from the EPIC array was imported into R 3.5.1 for normalization, background correction, and filtering. Beta values ranging from 0 (no methylation) to 1 (complete methylation) were calculated as a measure of methylation level and processed through the established support vector machine (SVM) classification algorithm for EpiSign^TM^ disorders. The EpiSign Knowledge Database composed of thousands of methylation profiles from reference disorder-specific and unaffected control cohorts was utilized by the classifier to generate disorder-specific methylation variant pathogenicity (MVP) scores. MVP scores are a measure of prediction confidence for each disorder, ranging from 0 (discordant) to 1 (highly concordant). For patients with full pathogenic mutations, a positive EpiSign^TM^ classification typically involves MVP scores greater than 0.5 in combination with concordant hierarchical clustering and multidimensional scaling.

## Results

The child came to medical attention at 7 months of age with marked failure to thrive and significant short stature (−3.5SD; WHO Growth Chart for Canada). Upper GI and gastric emptying studies were normal, with exception of gastroesophageal reflux, and a G-tube was placed. Despite the G-tube, his weight continued to decrease below the 3^rd^ percentile (at −2.75SD at last assessment). His stature has ranged from −4.22SD to −3.19SD, while head circumference has consistently tracked in the normal range between 10–15^th^ percentile.

The proband was also noted to have global developmental delay during his admission (at 7–8 months of age). His developmental milestones were considered appropriate for an individual of 3–4 months of age. He was not able to tripod sit or sit with support and was not able to reach out and grab objects. He vocalized when alone, however he would not respond to his name. The patient at 4 years of age continues to experience significant delays in all areas of communication. He is able to speak in short sentences though he is described as “very difficult to understand” by non-family members. The patient attends speech and language therapy. There have been improvements in his gross motor skills and he is now able to run but with frequent falls.

There is limited information available with regards to the proband’s prenatal and early life history. He was born to a G2P1 mother and there was documented alcohol exposure during the pregnancy. The proband was born at 35 weeks and 3 days gestation with a reported undescended testis at birth with no other issues.

On review of systems, the patient’s strabismus was noted at 6 months of age, requiring surgery at 2 years of age. Audiology assessment was normal. The proband has no history of regression or seizures.

The patient was placed in the care of his adoptive parents at 1 week of age. Both biological parents are reported to have issues with life skills and his biological mother was reported to have a mild intellectual disability and behavioral concerns.

**FIGURE 1:**
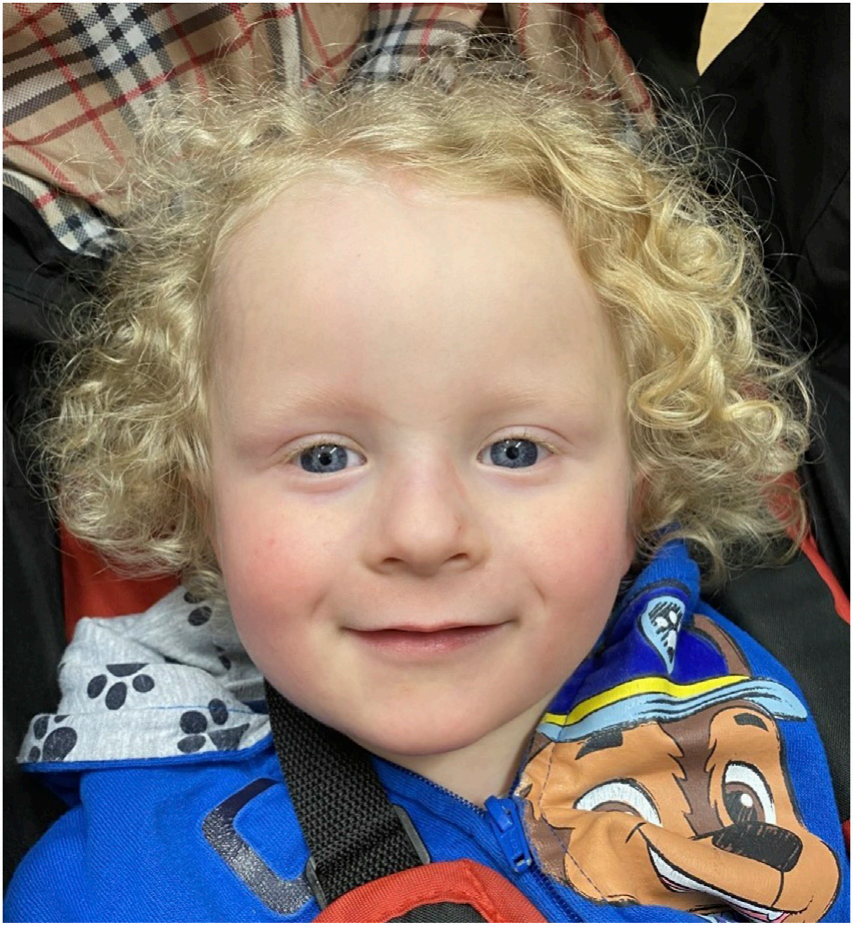
Clinical features of the patient described in this report at 4 years of age. The child showed fine, thin, curly hair with high anterior hairline and broad forehead, sparse eyebrows, epicanthus to left palpebral fissure and a thin upper vermillion.

The proband’s facial dysmorphisms were described at 7 months of age as fine, thin, curly hair with high anterior hairline and broad forehead, sparse eyebrows, epicanthus to left palpebral fissure and a thin upper vermillion. Figure 1 shows the proband at 4 years of age.

Chromosomal microarray and Russel-Silver syndrome testing were normal. Clinical whole-exome sequencing was performed by a commercial laboratory (2019) and was non-diagnostic. A re-analysis was requested and performed by the same laboratory (2021). As there was no genetic diagnosis, the family subsequently provided informed consent to be enrolled in the Care4Rare Canada research project. Reanalysis of the singleton exome sequencing data, on a research basis (2021), also did not provide a diagnosis.

**FIGURE 2:**
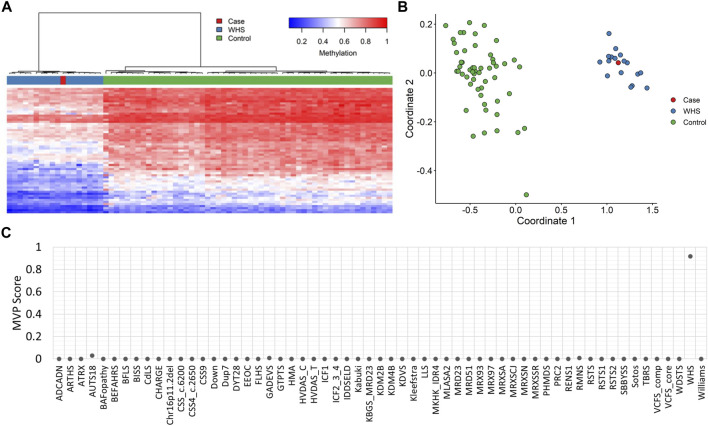
EpiSignTM DNA methylation analysis of peripheral blood from a patient with a negative microarray **(A)** Hierarchical clustering and **(B)** multidimensional scaling plots indicate that the patient (purple) has a DNA methylation signature similar to subjects with a confirmed WHS episignature (red) and distinct from controls (green). Each row of the heatmap represents one CpG probe on the DNA methylation array, and each column represents one individual’s sample. The heatmap color scale from blue to red represents the DNA methylation level (beta value) from 0 (no methylation) to 1 (fully methylated). **(C)** MVP score, a multiclass supervised classification system capable of discerning between multiple episignatures by generating a probability score for each episignature. The elevated patient score for WHS compared to other syndromes suggests an episignature similar to the WHS reference signature.

EpiSign^TM^ analysis performed at London Health Sciences Centre, as part of a national study assessing EpiSign^TM^ clinical utility called EpiSign-CAN (https://genomecanada.ca/project/beyond-genomics-assessing-improvement-diagnosis-rare-diseases-using-clinical-epigenomics-canada/), revealed an episignature consistent with WHS (Figure 2). Episignature detected for this patient was concordant with a methylation signature observed in patients with WHS syndrome, including Euclidean clustering (Figure 2A) and multidimensional scaling (Figure 2B). Additionally, MVP score for this patient was high for WHS, further suggesting a match with the WHS methylation signature (Figure 2C). Further review of the exome data within the deletion region for WHS highlighted a frameshift variant in *NSD2*, NM_001042424.3:c.4028del:p.Pro1343GlnfsTer49.

## Discussion

The patient reported in this study presented with syndromic short stature and developmental delay but remained without a molecular diagnosis despite comprehensive analysis through microarray and exome sequencing. DNA methylation episignature analysis provides another tool in the molecular diagnostic pathway—looking beyond genomic sequence or copy number variant assessment. Episignatures represent a functional consequence of a genetic defect in patients with neurodevelopmental disorders. Disruption of whole genome methylation in peripheral blood in those with genetic disorders has been well characterized and the resulting DNA methylation signatures act as sensitive and specific biomarkers for patients with ambiguous clinical and/or genetic findings (Aref-Eshghi et al., 2017; Aref-Eshghi et al., 2018; Aref-Eshghi et al., 2019a; Aref-Eshghi et al., 2020; Sadikovic et al., 2021; Kerkhof et al., 2022; Levy et al., 2022). In this case, EpiSign^TM^ analysis was used to screen for several disorders within the differential diagnosis based on the patient’s clinical presentation. The observed WHS episignature for this patient was curious given the negative chromosomal microarray and absence of a chromosome 4p deletion. The exome data obtained for the WHS critical regions was subsequently reassessed in further detail, and indeed a previously missed frameshift variant was observed in *NSD2*. Loss of function variants in this gene are associated with RAUST that was consistent with the patient’s presentation.

The molecular diagnosis was missed by the singleton exome sequencing performed by both a commercial laboratory and later a research laboratory. A key reason was likely the emerging “disease gene” status of NSD2. The definitive paper describing this syndrome was published in 2021 (Zanoni et al., 2021) and the exome sequence was initially performed in 2019. Other contributing factors to the missed diagnosis may have been the lack of parental samples sequenced and the early annotation of *NDS2* (originally called *WHSC1* or Wolff-Hirschhorn Syndrome Candidate gene). Without the methylation signature, the variant may have taken far longer to identify; however, we appreciate that if the exome sequencing was performed today the likelihood of missing the frameshift variant in *NSD2*, in a child with mild delays and global developmental delays, would be low.

Individuals with RAUST share similar facial gestalt including a triangular face, broad forehead, high anterior hairline, deeply set eyes, broad arched and laterally sparse brows, periorbital hyperpigmentation, a thin and elevated nasal bridge, smooth short philtrum among other features (Zanoni et al., 2021). More than half of these individuals exhibit developmental delay, intrauterine growth restriction, hypotonia, feeding difficulties, failure to thrive, and speech delay. Height and head circumference measurements below the 5^th^ percentile (Zanoni et al., 2021). These features do also overlap with WHS, however, these patients lack the characteristic “Greek warrior helmet” facial features of WHS, in addition to seizures, orofacial clefts, as well as a higher frequency of genital, cardiac and renal malformations (Zanoni et al., 2021). The degree of severity of intellectual disability in the published *NSD2* cohort is also less than WHS patients with the more common, larger 4p deletions (Zanoni et al., 2021). Supplementary Table S1 lists phenotypic features of WHS and RAUST syndrome, as well as whether these features are present in the proband.

Preliminary data (unpublished) has demonstrated that patients with *NSD2* truncating variants have been shown to match the established WHS episignature, however further assessment of concordance between episignatures resulting from truncating and missense variants within *NSD2* relative to the WHS episignature is required. Additionally, exploration of a *NSD2-*specific episignature would allow for assessment of similarity and differences in DNA methylation changes between syndromes. Furthermore, phenotypic, genotypic and episignature correlation studies would contribute to a better understanding of WHS and RAUST pathophysiology. An example where this approach was applied is another chromosome deletion syndrome with a defined episignature, Phelan-McDermid syndrome (OMIM# 606232). This study compared the methylation profile of patients with large or small deletions and established an episignature for patients with large deletions (Schenkel et al., 2021). This allowed for identification of a critical region that was present in all those with large deletions demonstrating the shared episignature but absent in the small deletion patients who did not match the episignature (Schenkel et al., 2021). This critical region included only one fully contained protein-coding gene: *BRD1 (*bromodomain-containing protein 1; OMIM #604589). BRD1 is a component of a histone acetyltransferase complex that can acetylate histone H3 through interactions with other chromatin remodeling proteins and histone modifiers (Christensen et al., 2012; Fryland et al., 2016). Given the protein’s function in the epigenetic machinery, it presents as a candidate that may impact whole genome methylation and ultimately gene expression.

Our report provides evidence to the role DNA methylation in the molecular etiology of *NSD2* related neurodevelopmental disorders. A truncating variant in *NSD2* presenting with very similar methylation pattern to WHS patients provides functional evidence that though WHS likely involves multigenic pathogenesis, loss of NSD2 contributes in part to epigenetic changes observed in WHS. These changes may influence gene expression and ultimately development, however further studies, including DNA expression assessment, are required. This report provides a key example of the clinical utility of EpiSign^TM^. The concurrent screening of 65 syndromes provides invaluable information that can guide the search for genetic variants as well as the interpretation of whole exome sequencing output. This report highlights the power of a multi-omics approach in the clinic and how this can improve diagnostic yield for patients.

## Data Availability

The datasets presented in this article are not readily available because the data that support the findings of this study were obtained from clinical laboratories and a research study. Restrictions apply to the availability of these data, which are not publicly available due to privacy and ethical restrictions. Requests to access the datasets should be directed to BS, bekim.sadikovic@lhsc.on.ca.
