# Supplementary material for: Genetically unresolved case of Rauch-Steindl syndrome diagnosed by its wolf-hirschhorn associated DNA methylation episignature
Source: Front Cell Dev Biol. 2022 Dec 15;10:1022683. doi: 10.3389/fcell.2022.1022683 (PMC9800036; doi:10.3389/fcell.2022.1022683)
Supplement: Supplementary file 1 [file Table1.DOCX]

**Supplemental Table 1.** Comparison of Wolff-Hirschhorn Syndrome and Rauch-Steindl syndrome

| **Phenotypic features** | **Wolff-Hirschhorn**  **Syndrome*** | **Rauch-Steindl syndrome**** | **Proband** |
| --- | --- | --- | --- |
| ***Prenatal*** | | | |
| Intrauterine growth restriction | + | + | Not known |
| Decreased fetal movement | + | - | Not known |
| ***Measurements at birth*** | | | |
| Birth weight | Low birth weight | Low birth weight | Not known |
| Birth length | SGA | SGA | Not known |
| Birth head circumference | Microcephaly | Microcephaly | Not known |
| ***Development and behaviour*** | | | |
| Global developmental delay | +  (significant delays) | +  (mild) | +  (Mild) |
| Intellectual disability | +  (Severe/profound) | +  (Mild) | +  (Mild) |
| Behavioural abnormalities | +  (Psychotic behaviours) | +  (Aggression, anxiety, ADHD, autistic features) | Happy demeanor |
| ***Facial features*** | | | |
| “Greek helmet” appearance | + | - | - |
| Forehead | Broad | Broad | Broad |
| Eyebrows | Arched | Normal | Sparse |
| Hypertelorism | + | + | No |
| Epicanthal folds | + | + | + |
| Ears | Poorly formed | Pronounced | Normal |
| Down turned corners of mouth | + | - | - |
| Cleft lip/palate | + | + | Absent |
| Micrognathia | + | + | + |
| Other | NA | NA | Fine, thin, curly hair and a thin upper vermillion border |
| ***Post-natal growth*** | | | |
| Failure to thrive | + | + | +  Weight (-2.75SD) |
| Postnatal microcephaly | + | + | 10-15%  (low-normal) |
| Postnatal growth restriction | + | + | Short Stature  (-4.22SD to -3.19SD) |
| ***Neurological issues*** | | | |
| Hypotonia | + | + | + |
| Seizures | +  (90%; onset <1 year) | Rare | - |
| Structural brain malformations | + | + | - |
| ***Ophthalmological issues*** | | | |
| Refractive errors | + | + | - |
| Strabismus | + | + | + |
| Coloboma | + | - | - |
| Optic nerve anomalies | + | Rare | - |
| ***Skeletal issues*** | | | |
| Kyphosis/scoliosis | + | - | - |
| Clubfeet | + | - | - |
| Split hand deformity | + | - | - |
| Other | Accessory/fused ribs | Clinodactyly | - |
| ***Cardiac issues*** | | | |
| ASD | + | - | - |
| VSD | + | - | - |
| ***Renal/Genitourinary issues*** | | | |
| Renal hypoplasia or malformation | + | + | - |
| Hypospadias | + | + | - |
| Cryptorchidism | + | + | + |
| Absent uterus | + | - | NA |

*taken from Battaglia et 2008; **Weil et al 2022. Definitions: SGA, small for gestational age; ADHD, attention deficit, hyperactivity disorder; ASD atrial septal defect; VSD ventricular septal defect
